# Identifying therapeutic target genes for migraine by systematic druggable genome-wide Mendelian randomization

**DOI:** 10.1186/s10194-024-01805-3

**Published:** 2024-06-12

**Authors:** Chengcheng Zhang, Yiwei He, Lu Liu

**Affiliations:** 1 Department of Acupuncture and Moxibustion, Beijing Hospital of Traditional Chinese Medicine, Capital Medical University, Beijing Key Laboratory of Acupuncture Neuromodulation, No. 23, Meishuguan Houjie, Beijing, 100010 China; 2 State Key Laboratory of Networking and Switching Technology, Beijing University of Posts and Telecommunications, Beijing, 100876 China

**Keywords:** Migraine, Mendelian randomization, Druggable target genes

## Abstract

**Background:**

Currently, the treatment and prevention of migraine remain highly challenging. Mendelian randomization (MR) has been widely used to explore novel therapeutic targets. Therefore, we performed a systematic druggable genome-wide MR to explore the potential therapeutic targets for migraine.

**Methods:**

We obtained data on druggable genes and screened for genes within brain expression quantitative trait locis (eQTLs) and blood eQTLs, which were then subjected to two-sample MR analysis and colocalization analysis with migraine genome-wide association studies data to identify genes highly associated with migraine. In addition, phenome-wide research, enrichment analysis, protein network construction, drug prediction, and molecular docking were performed to provide valuable guidance for the development of more effective and targeted therapeutic drugs.

**Results:**

We identified 21 druggable genes significantly associated with migraine (BRPF3, CBFB, CDK4, CHD4, DDIT4, EP300, EPHA5, FGFRL1, FXN, HMGCR, HVCN1, KCNK5, MRGPRE, NLGN2, NR1D1, PLXNB1, TGFB1, TGFB3, THRA, TLN1 and TP53), two of which were significant in both blood and brain (HMGCR and TGFB3). The results of phenome-wide research showed that HMGCR was highly correlated with low-density lipoprotein, and TGFB3 was primarily associated with insulin-like growth factor 1 levels.

**Conclusions:**

This study utilized MR and colocalization analysis to identify 21 potential drug targets for migraine, two of which were significant in both blood and brain. These findings provide promising leads for more effective migraine treatments, potentially reducing drug development costs.

**Supplementary Information:**

The online version contains supplementary material available at 10.1186/s10194-024-01805-3.

## Background

Migraine is a prevalent chronic disease characterized by recurring headaches that are typically unilateral and throbbing, ranging from moderate to severe intensity, and often accompanied by nausea, vomiting, sensitivity to light, among other symptoms [1]. Migraine is recognized as the second most disabling condition globally, creating substantial challenges for those affected and also placing a considerable strain on society overall [2]. Genetic factors play a substantial role in migraine, with its heritability estimated to be as high as 57% [3].

Currently, the treatment and prevention of migraine remain highly challenging. Although new drugs (e.g. targeting the calcitonin gene-related peptide, namely CGRP) have been developed, offering significant benefits to migraine sufferers, there are still many issues, such as side effects and less than ideal response rates [4]. Therefore, it is necessary to continue exploring potential therapeutic targets for migraine treatment. Integrating genetics into drug development may provide a novel approach. While genome-wide association studies (GWAS) are very effective in identifying single nucleotide polymorphisms (SNPs) associated with the risk of migraine [5], the GWAS method does not clearly and directly identify the causative genes or drive drug development without substantial downstream analyses [6, 7].

Mendelian randomization (MR) is a method that utilizes genetic variation as instrumental variables (IVs) to uncover a causal connection between an exposure and an outcome [8]. MR analysis has been widely applied to discover new therapeutic targets by integrating summarized data from disease GWAS and expression quantitative trait loci (eQTL) studies [9]. The eQTLs found in the genomic regions of druggable genes are always considered as proxies, since the expression levels of gene can be seen as a form of lifelong exposure. Therefore, we performed a systematic druggable genome-wide MR to explore the potential therapeutic targets for migraine. First, we obtained data on druggable genes and screened for genes within brain eQTLs and blood eQTLs, which were then subjected to two-sample MR analysis with migraine GWAS data to identify genes highly associated with migraine. Subsequently, we conducted colocalization analysis to ensure the robustness of our results. For significant genes both in blood and brain, the phenome-wide research was conducted to explore the relationship between shared potential therapeutic targets and other characteristics. In addition, enrichment analysis, protein network construction, drug prediction, and molecular docking were performed for all significant genes to provide valuable guidance for the development of more effective and targeted therapeutic drugs.

## Methods

**Fig. 1:**
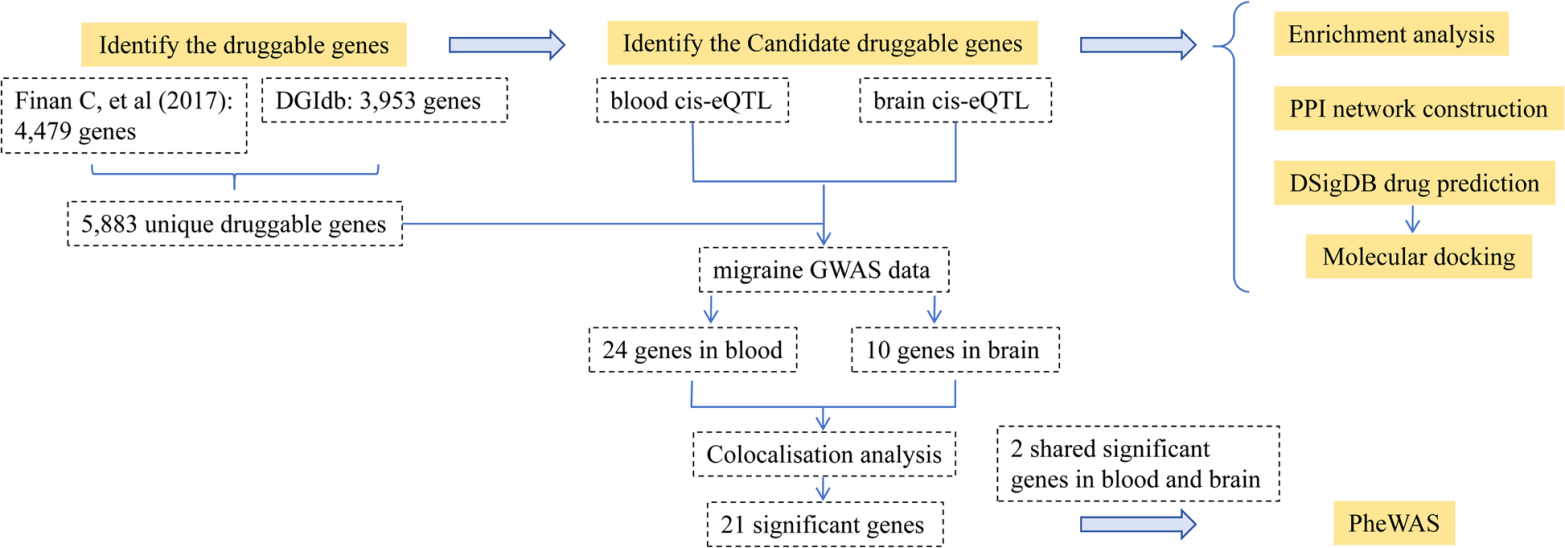
Overview of this study design. DGIdb: Drug-Gene Interaction Database; eQTL: expression quantitative trait loci; GWAS: genome-wide association studies; PheWAS: Phenome-wide association study; PPI: protein–protein interaction; DSigDB: Drug Signatures Database

The overview of this study is presented in Fig. 1.

### Druggable genes

Druggable genes were sourced from the Drug-Gene Interaction Database (DGIdb, https://www.dgidb.org/) [10] and a comprehensive review [11]. The DGIdb offers insights into drug-gene interactions and the potential for druggability. We accessed the 'Categories Data' from DGIdb, which was updated in February 2022. Additionally, we utilized a list of druggable genes provided in a review authored by Finan et al. [11]. By consolidating druggable genes from two sources, a broader range of druggable genes can be obtained, which have already been applied in previous study [12].

### eQTL datasets

The blood eQTL dataset was sourced from eQTLGen (https://eqtlgen.org/) [13], which provided cis-eQTLs for 16,987 genes derived from 31,684 blood samples collected from healthy individuals of European ancestry (Table 1). We acquired cis-eQTL results that were fully significant (with a false discovery rate (FDR) less than 0.05) along with information on allele frequencies. We obtained the brain eQTL data from the PsychENCODE consortia (http://resource.psychencode.org) [14], encompassing 1,387 samples from the prefrontal cortex, primarily of European descent (Table 1). We downloaded all significant eQTLs (with FDR less than 0.05) for genes that exhibited an expression level greater than 0.1 fragments per kilobase per million mapped fragments in at least 10 samples, along with complete SNP information.

**Table 1:** The details of eQTL and GWAS used in the study

Dataset	Sample size	Ancestry	Consortium	Web link
The blood cis-eQTL	31,684	European	eQTLGen	https://eqtlgen.org/
The brain cis-eQTL	1,387	Primarily European	PsychENCODE	http://resource.psychencode.org
Migraine GWAS	589,356	European	IHGC	https://www.nature.com/articles/s41588-021-00990-0

### Migraine GWAS dataset

In this study, the summary statistics data for migraine were obtained from a meta-analysis of GWAS conducted by the International Headache Genetics Consortium (IHGC) in 2022 [5]. To address privacy concerns related to participants in the 23andMe cohort, the GWAS summary statistics data used in this study did not include samples from the 23andMe cohort. The summary data comprised 589,356 individuals of European ancestry, with 48,975 cases and 540,381 controls (Table 1).

### Mendelian randomization analysis

MR analyses were conducted using the 'TwoSampleMR' package (version 0.5.7) [15] in R. We chose the eQTLs of the drug genome as the exposure data. For constructing IVs, SNPs with a FDR below 0.05 and located within ± 100 kb of the transcriptional start site (TSS) of each gene were selected. These SNPs were subsequently clumped at an r^2^ less than 0.001 using European samples from the 1000 Genomes Project [16]. The R package 'phenoscanner' [17] (version 1.0) was employed to identify phenotypes related to the IVs. Additionally, we excluded SNPs that were directly associated with migraine and the trait directly linked to migraine, namely headache. We harmonised and conducted MR analyses on the filtered SNPs. When only one SNP was available for analysis, we use the Wald ratio method to perform MR estimation. When multiple SNPs were available, MR analysis was performed using the inverse-variance weighted (IVW) method with random effects [18]. We used Cochran's Q test to assess heterogeneity among the individual causal effects of the SNPs [19]. Additionally, MR Egger's intercept was utilized to evaluate SNP pleiotropy [20]. *P*-values were adjusted by FDR, and 0.05 was considered as the significant threshold. Additionally, we selected target genes associated with commonly used medications for migraine and compared their MR results with those of significantly druggable genes.

### Colocalization analysis

Sometimes, a single SNP is located in the regions of two or more genes. In such cases, its impact on a disease (here, migraine) is influenced by a mix of different genes. Colocalization analysis was used to confirm the potential shared causal genetic variations in physical location between migraine and eQTLs. We separately filtered SNPs located within ± 100 kb from each migraine risk gene's TSS from migraine GWAS data, blood eQTL data, and brain eQTL data. The probability that a given SNP is associated with migraine is denoted as P1, the probability that a given SNP is a significant eQTL is denoted as P2, and the probability that a given SNP is both associated with migraine and is an eQTL result is denoted as P12. All probabilities were set to default values (P1 = 1 × 10^−4^, P2 = 1 × 10^−4^, and P12 = 1 × 10^−5^) [21]. We used posterior probabilities (PP) to quantify the support for all hypotheses, which are identified as PPH0 through PPH4: PPH0, not associated with any trait; PPH1, related to gene expression but not associated with migraine risk; PPH2, associated with migraine risk but not related to gene expression; PPH3, associated with both migraine risk and gene expression, with clear causal variation; and PPH4, associated with both migraine risk and gene expression, with a common causal variant. Given the limited capacity of colocalization analysis, we restricted our subsequent analyses to genes where PPH4 was greater than or equal to 0.75. Colocalization analysis was conducted using the R package 'coloc' (version 5.2.3).

### Phenome-wide association analysis

We used the IEU OpenGWAS Project (https://gwas.mrcieu.ac.uk/phewas/) [15] to obtain the phenome-wide association study (PheWAS) data of SNPs corresponding to druggable genes that were significant in both blood and brain following colocalization analysis.

### Enrichment analysis

To explore the functionals' characteristics and biological relevance of predetermined prospective druggable genes, the R package 'clusterProfiler' (version 4.10.1) [22] was used for Gene Ontology (GO) and Kyoto Encyclopedia of Genes and Genomes (KEGG) enrichment studies. GO includes three terms: Biological Process (BP), Molecular Function (MF), and Cellular Component (CC). KEGG pathways can provide information about metabolic pathways.

### Protein–protein interaction network construction

The protein–protein interaction (PPI) networks can visually display the relationships between protein interactions of significant druggable genes. We constructed PPI networks using the STRING (https://string-db.org/) s' with a confidence score threshold of 0.4 as the minimum required interaction score, while all other parameters were maintained at their default settings [23].

### Candidate drug prediction

Drug Signatures Database (DSigDB, http://dsigdb.tanlab.org/DSigDBv1.0/) [24] is a sizable database with 22,527 gene sets and 17,389 unique compounds spanning 19,531 genes. We uploaded previously identified significant druggable genes to DSigDB to predict candidate drugs and evaluate the pharmacological activity of target genes.

### Molecular docking

We conducted molecular docking to assess the binding energies and interaction patterns between candidate drugs and their targets. By identifying ligands that exhibit high binding affinity and beneficial interaction patterns, we are able to prioritize drug targets for additional experimental validation and refine the design of prospective candidate drugs. Drug structural data were sourced from the PubChem Compound Database (https://pubchem.ncbi.nlm.nih.gov/) [25] and downloaded in SDF format, then converted to pdb format using OpenBabel 2.4.1. Protein structural data were downloaded from the Protein Data Bank (PDB, http://www.rcsb.org/). The top five important drugs and the proteins encoded by the respective target genes were subjected to molecular docking using the computerized protein–ligand docking software AutoDock 4.2.6 (http://autodock.scripps.edu/) [26], and the results were visualized using PyMol 3.0.2 (https://www.pymol.org/). The final structures of six proteins and four drugs were obtained.

## Results

### Druggable genome

We obtained 3,953 druggable genes from the DGIdb (Table S1). Additionally, we acquired 4,463 druggable genes from previous reviews (Table S2) [11]. After integrating the data, we obtained 5,883 unique druggable genes named by the Human Genome Organisation Gene Nomenclature Committee for subsequent analysis (Table S3).

### Candidate druggable genes

**Fig. 2:**
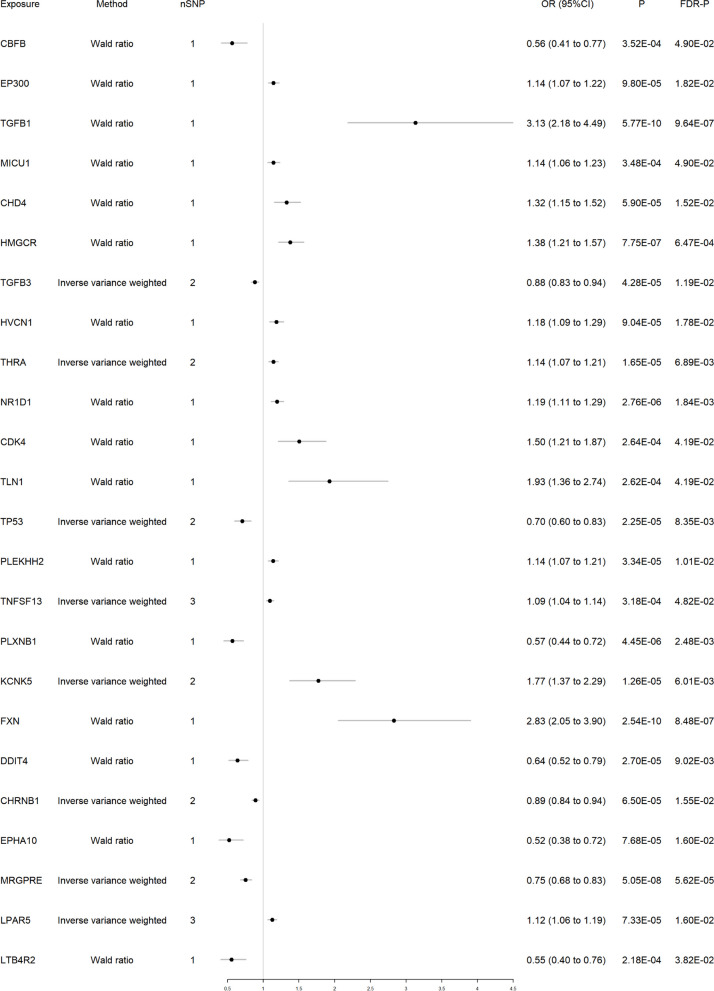
Forest plot of 24 significant genes associated with migraine from blood

**Fig. 3:**
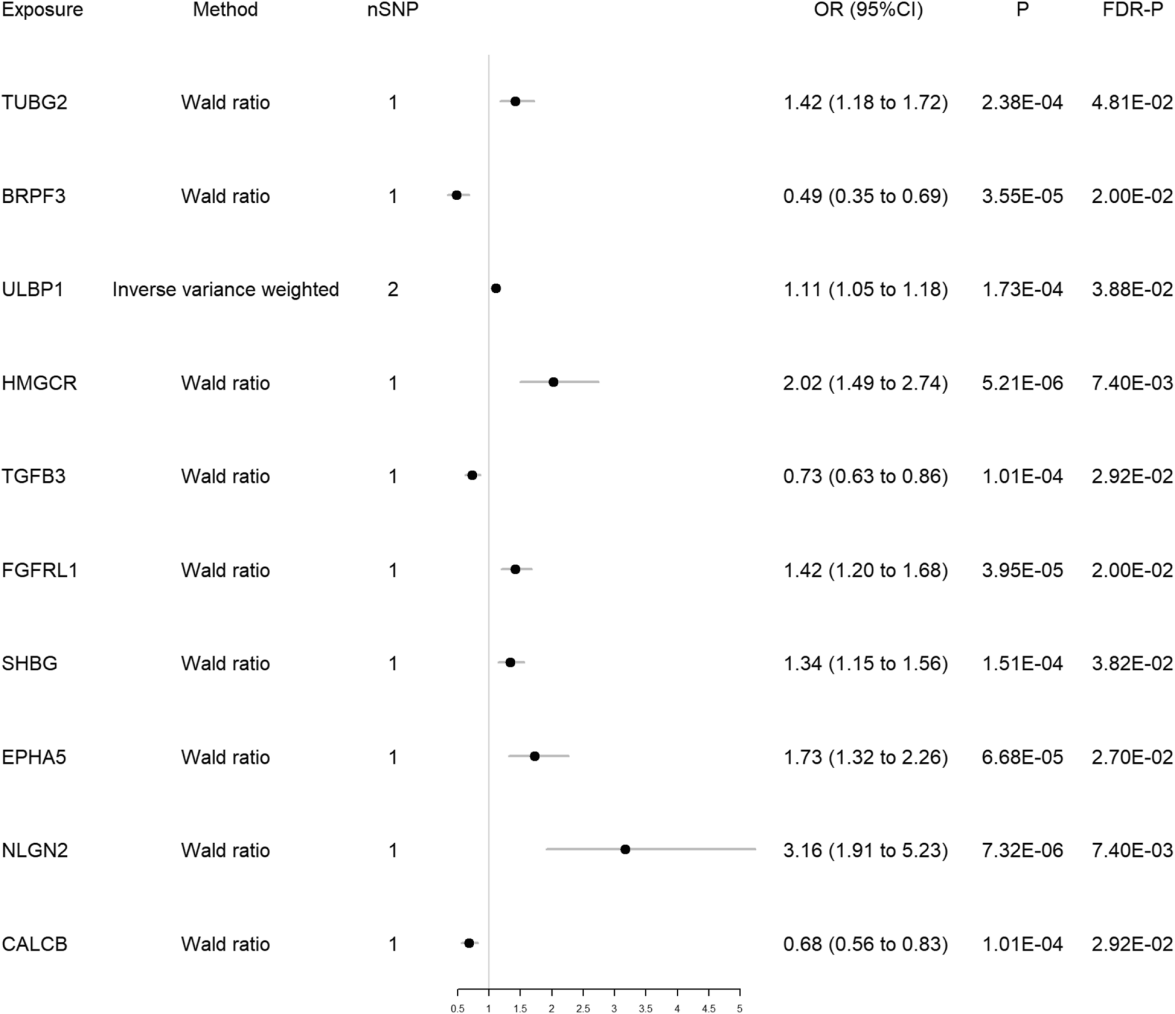
Forest plot of 10 significant genes associated with migraine from brain

After intersecting eQTLs from blood and brain tissue with druggable genes respectively, the blood eQTLs contained 3,460 gene symbols, while the brain eQTLs had 2,624 gene symbols. We performed MR analysis and identified 24 significant genes associated with migraine from blood and 10 from brain tissue (Figs. 2 and 3). Among them, two genes, HMGCR and TGFB3, reached significance in both blood (HMGCR OR 1.38 and TGFB3 OR 0.88) and brain tissues (HMGCR OR 2.02 and TGFB3 OR 0.73). Detailed results for the significant IVs and full results of MR are available in the Table S4-S6.

**Fig. 4:**
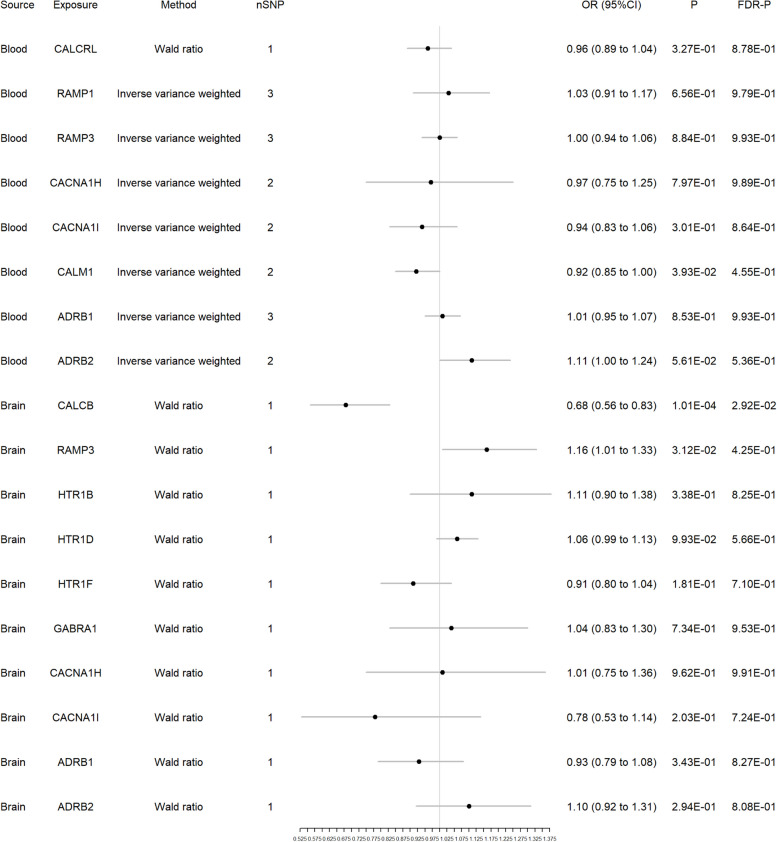
Forest plot of 13 genes associated with commonly used medications for migraine from blood and brain

We selected target genes associated with commonly used medications for migraine as comparisons for our study results [27]. These include CGRP-related gene (CALCB, CALCRL, RAMP1 and RAMP3), genes related to 5-hydroxytryptamine (5-HT) receptors targeted by ergot alkaloids, triptans, and ditans (HTR1B, HTR1D, HTR1F), γ-aminobutyric acid (GABA) receptor-related genes targeted by topiramate (GABRA1), calcium ion channel-related genes targeted by flunarizine (CACNA1H, CACNA1I, CALM1), and genes related to β-adrenoceptor targeted by propranolol (ADRB1, ADRB2). Among these genes (Fig. 4), CALM1 showed significant association with migraine in blood eQTL, but it lost significance after FDR correction (OR 0.92, *P* = 0.039, FDR-P = 0.455). In brain eQTL, CALCB and RAMP3 showed correlation with migraine, and after FDR correction, CALCB still maintained significance (CALCB: OR 0.68, *P* = 0.0001, FDR-P = 0.029; RAMP3: OR 1.16, *P* = 0.031, FDR-P = 0.425).

### Colocalization analysis

The results indicated that, of the previously identified 24 significant genes from blood, 17 had a PPH4 greater than 0.75. Among the 10 significant genes from brain, 6 had a PPH4 greater than 0.75. HMGCR and TGFB3 showed significant colocalization results in both blood and brain tissues (Table 2, Table 3 and Table S7).

**Table 2:** Colocalization results of 17 significant genes from blood

Gene	PPH0	PPH1	PPH2	PPH3	PPH4
CBFB	0.000	0.000	0.179	0.042	0.778
EP300	0.000	0.000	0.100	0.052	0.848
TGFB1	0.000	0.001	0.000	0.015	0.985
CHD4	0.000	0.000	0.057	0.055	0.888
HMGCR	0.000	0.000	0.001	0.029	0.970
TGFB3	0.000	0.000	0.055	0.000	0.945
HVCN1	0.000	0.000	0.063	0.075	0.862
THRA	0.000	0.000	0.003	0.002	0.995
NR1D1	0.000	0.000	0.003	0.002	0.995
CDK4	0.000	0.000	0.134	0.001	0.865
TLN1	0.003	0.000	0.116	0.007	0.874
TP53	0.000	0.000	0.011	0.003	0.986
PLXNB1	0.000	0.000	0.007	0.093	0.900
KCNK5	0.000	0.000	0.070	0.026	0.905
FXN	0.000	0.000	0.000	0.003	0.997
DDIT4	0.000	0.000	0.035	0.023	0.943
MRGPRE	0.000	0.000	0.000	0.008	0.992

PPH0-PPH4 represent the posterior probabilities of different hypotheses, and PPH4 > 0.75 was considered as a significant colocalization result

**Table 3:** Colocalization results of 10 significant genes from brain

Gene	PPH0	PPH1	PPH2	PPH3	PPH4
BRPF3	0.067	0.008	0.035	0.003	0.888
HMGCR	0.110	0.087	0.011	0.008	0.785
TGFB3	0.045	0.001	0.052	0.000	0.902
FGFRL1	0.000	0.000	0.081	0.001	0.918
EPHA5	0.000	0.000	0.094	0.012	0.894
NLGN2	0.000	0.000	0.007	0.030	0.962

PPH0-PPH4 represent the posterior probabilities of different hypotheses, and PPH4 > 0.75 was considered as a significant colocalization result

### Phenome-wide association analysis

Due to the presence of the blood–brain barrier, compared to various components in the blood and other organs, brain tissue is more difficult to be affected by the action of drugs [28]. Therefore, we used the IEU OpenGWAS Project to obtain the PheWAS results of SNPs corresponding to HMGCR and TGFB3 from blood, rather than from brain tissue. The results showed that HMGCR was highly correlated with low-density lipoprotein (LDL), and TGFB3 was primarily associated with the level of insulin-like growth factor 1 (IGF1). The complete results are available in the Table S8-S9.

### Enrichment analysis

Through GO analysis of 21 potential targets, we found that these targets are primarily involved in BP such as regulation of protein secretion (GO: 0050708), response to hypoxia (GO: 0001666), negative regulation of carbohydrate metabolic processes (GO: 0045912), and the intrinsic apoptotic signaling pathway in response to DNA damage by p53 class mediator (GO: 0042771). The main MF include transcription coregulator binding (GO: 0001221) and chromatin DNA binding (GO: 0031490, Fig. 5). To explore the potential therapeutic pathways of migraine-associated significant druggable genes, KEGG analysis indicates that the target genes were primarily enriched in pathways such as Human T-cell leukemia virus 1 infection (hsa05166) and the Cell cycle (hsa04110, Fig. 6).

**Fig. 5:**
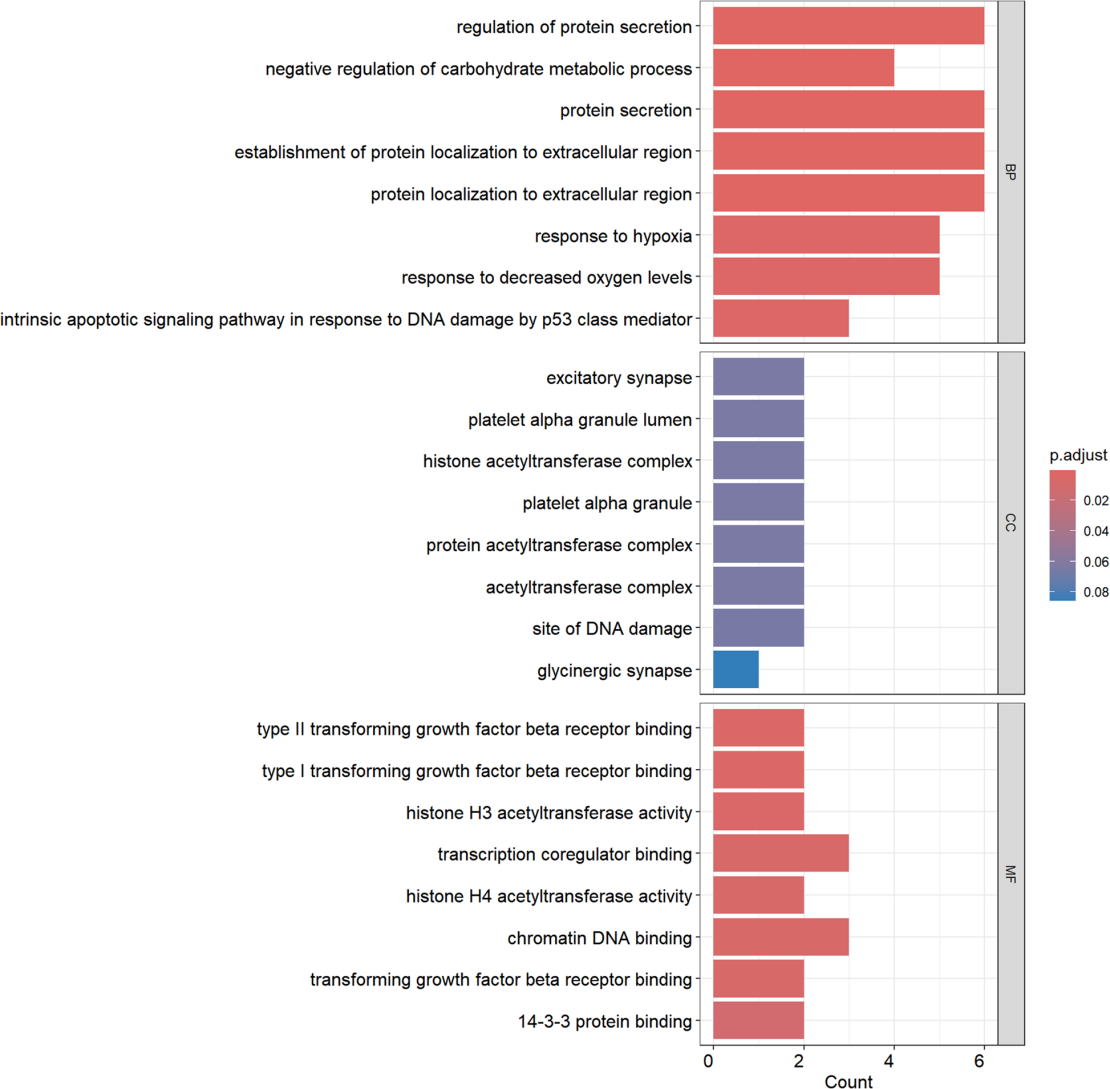
GO enrichment results for three terms

**Fig. 6:**
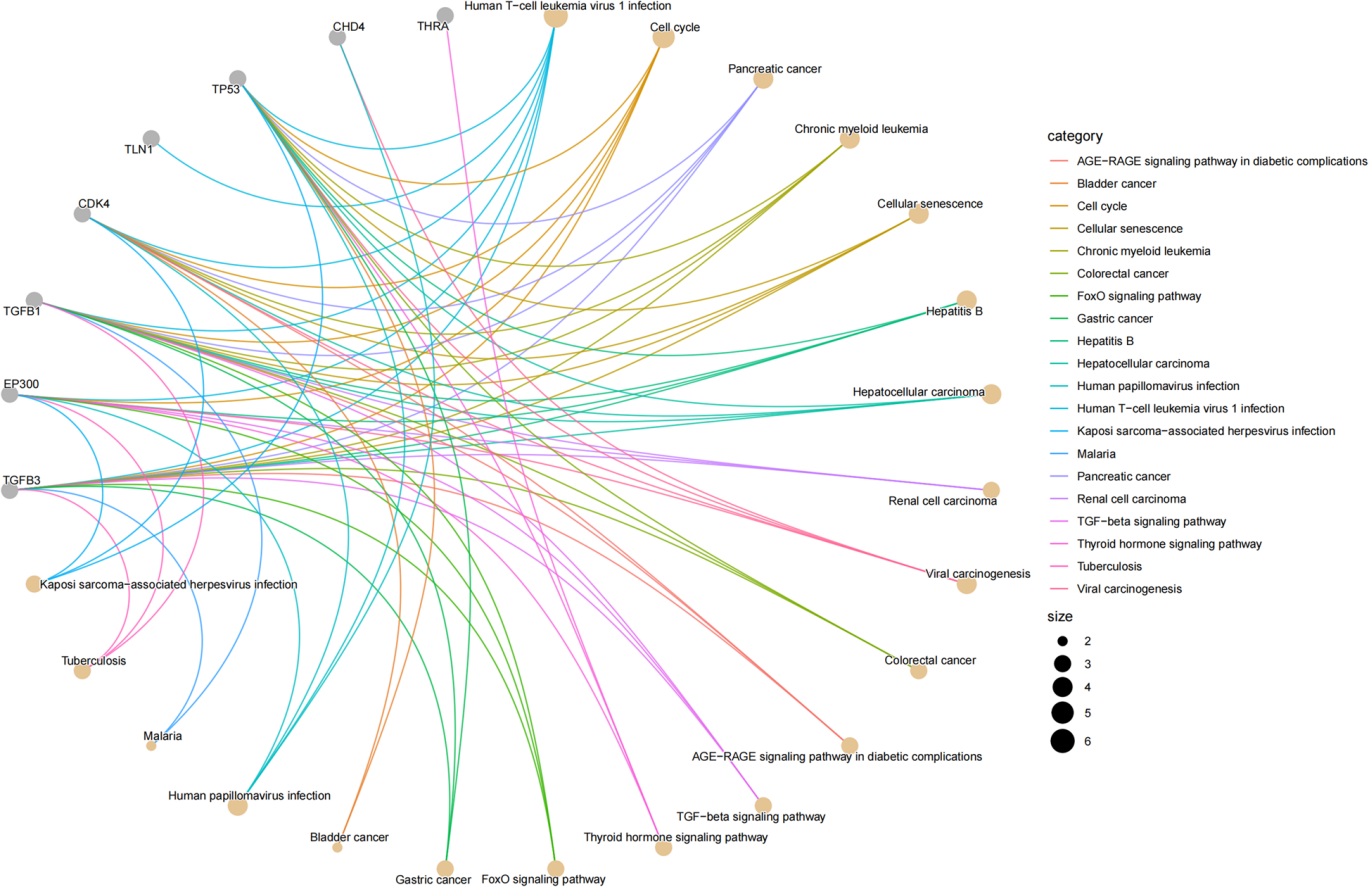
KEGG enrichment results

### Protein–protein interaction network construction

**Fig. 7:**
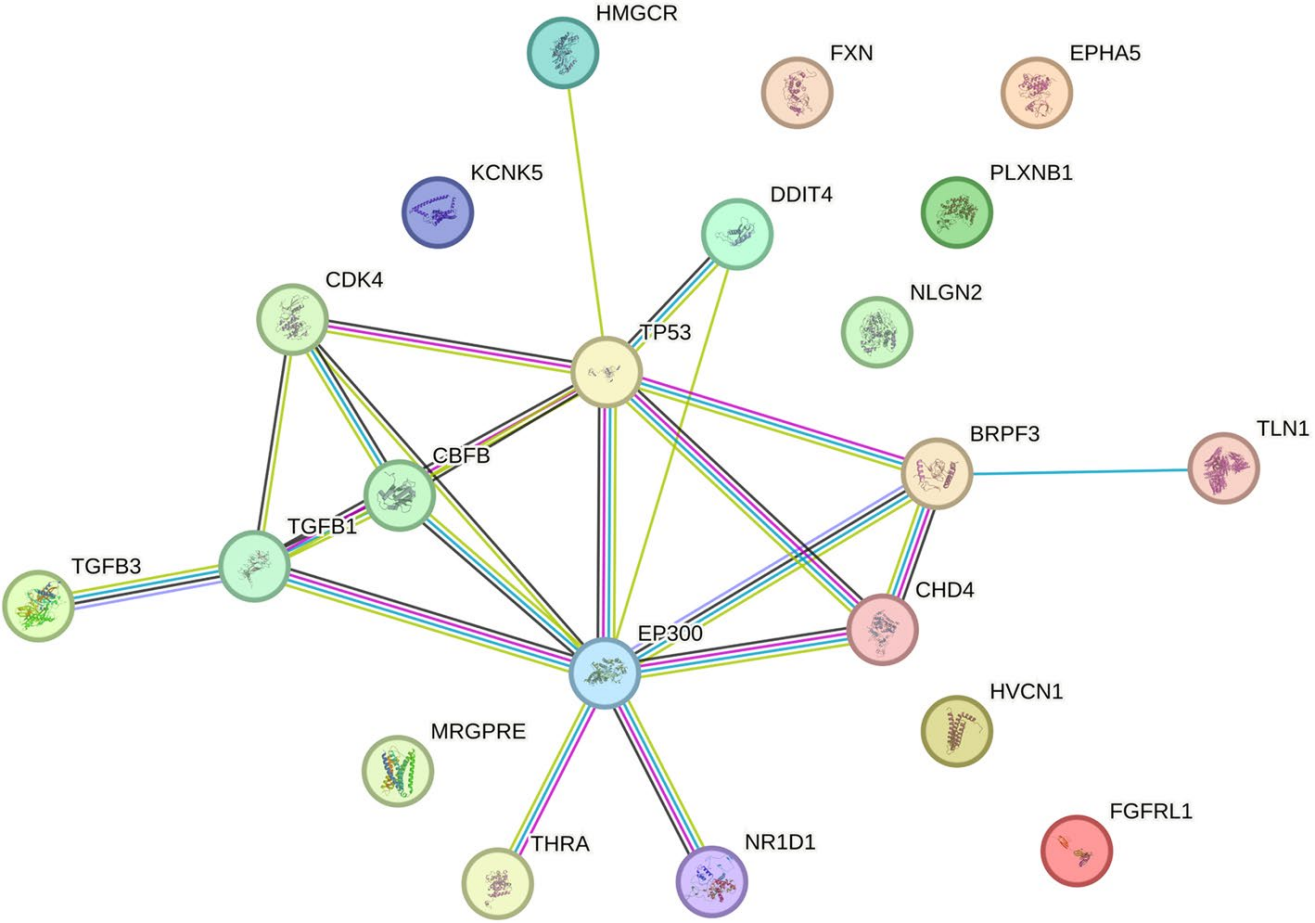
PPI network built with STRING

We loaded 21 drug target genes into the STRING database to create a PPI network. The results, shown in Fig. 7, displayed protein interaction pathways consisting of 21 nodes and 22 edges.

### Candidate drug prediction

We used DSigDB to predict potentially effective intervention drugs and listed the top 10 potential intervention drugs based on the adjusted *P*-values (Table 4). The results indicated that butyric acid (butyric acid CTD 00007353) and clofibrate (clofibrate CTD 00005684) were the two most significant drugs, connected respectively to TGFB1, TGFB3, EP300, TP53 and TGFB1, CDK4, HMGCR, TP53. Additionally, arsenenous acid (Arsenenous acid CTD 00000922) and dexamethasone (dexamethasone CTD 00005779) were associated with most of the significant druggable genes.

**Table 4:** Candidate drug predicted by DSigDB

Drug name	*P*-value	Adjusted *P*-value	Genes
butyric acid CTD 00007353	0.000	0.000	TGFB1; TGFB3; EP300; TP53
clofibrate CTD 00005684	0.000	0.000	TGFB1; CDK4; HMGCR; TP53
Sorafenib CTD 00004146	0.000	0.003	TGFB1; CDK4; DDIT4; TP53
Andrographolide CTD 00001445	0.000	0.005	TGFB1; CDK4; TP53
Arsenenous acid CTD 00000922	0.000	0.008	EPHA5; TGFB1; TGFB3; CDK4; DDIT4; EP300; TP53; FXN
Melatonin CTD 00006260	0.000	0.011	TGFB1; CDK4; TP53
Mehp CTD 00000849	0.000	0.011	EP300; HMGCR; TP53
dexamethasone CTD 00005779	0.000	0.012	TGFB1; TGFB3; EP300; NR1D1; TP53
NIMUSTINE CTD 00007067	0.000	0.013	FXN; TP53
Homocastasterone CTD 00002741	0.000	0.013	CDK4; TP53

### Molecular docking

**Fig. 8:**
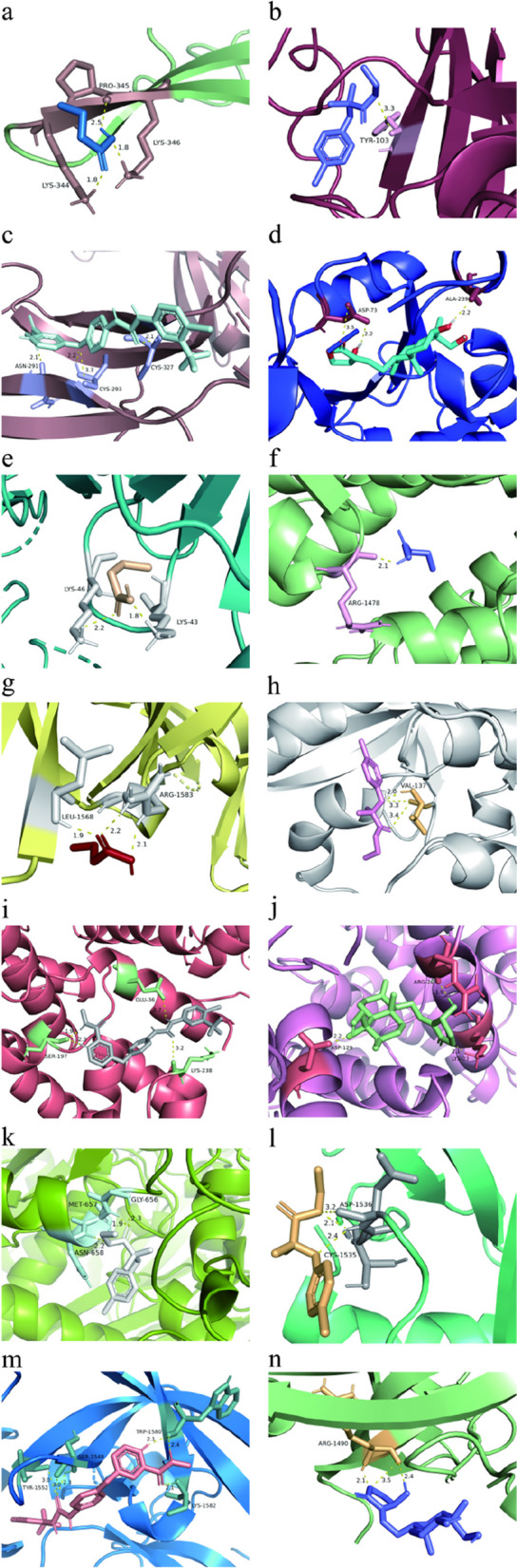
Molecular docking results of available proteins and drugs. **a** TGFB1 docking butyric acid, **b** TGFB1 docking clofibrate, **c** TGFB1 docking Sorafenib, **d** TGFB1 docking Andrographolide, **e** TGFB3 docking butyric acid, **f** EP300 docking butyric acid, **g** TP53 docking butyric acid, **h** CDK4 docking clofibrate, **i** CDK4 docking Sorafenib, **j** CDK4 docking Andrographolide, **k** HMGCR docking clofibrate, **l** TP53 docking clofibrate, **m** TP53 docking Sorafenib, **n** TP53 docking Andrographolide

We used AutoDock 4.2.6 to analyze the binding sites and interactions between the top 5 candidate drugs and the proteins encoded by the corresponding genes, generating the binding energy for each interaction. We obtained 14 effective docking results between the proteins and drugs (Table 5). Docking amino acid residues and hydrogen bond lengths are shown in Fig. 8. Among these, the binding between CDK4 and andrographolide exhibited the lowest binding energy (-7.11 kcal/mol), indicating stable binding.

**Table 5:** Molecular docking results of available proteins and drugs

Target	PDB ID	Drug	PubChem ID	Binding energy (kcal/mol)
TGFB1	5VQP	butyric acid	264	-4.17
TGFB1	5VQP	clofibrate	2796	-5.9
TGFB1	5VQP	Sorafenib	216,239	-5.32
TGFB1	5VQP	Andrographolide	5,318,517	-4.93
TGFB3	8V52	butyric acid	264	-4.4
EP300	8GZC	butyric acid	264	-3.3
TP53	6MY0	butyric acid	264	-4.05
CDK4	2W96	clofibrate	2796	-5.74
CDK4	2W96	Sorafenib	216,239	-5.65
CDK4	2W96	Andrographolide	5,318,517	-7.11
HMGCR	1HW9	clofibrate	2796	-4.35
TP53	6MY0	clofibrate	2796	-5.05
TP53	6MY0	Sorafenib	216,239	-7.05
TP53	6MY0	Andrographolide	5,318,517	-6.05

The lower the Binding energy, the better the binding effect and the higher the affinity

## Discussion

This study integrated existing druggable gene targets with migraine GWAS data through MR and colocalization analysis, identifying 21 druggable genes significantly associated with migraine (BRPF3, CBFB, CDK4, CHD4, DDIT4, EP300, EPHA5, FGFRL1, FXN, HMGCR, HVCN1, KCNK5, MRGPRE, NLGN2, NR1D1, PLXNB1, TGFB1, TGFB3, THRA, TLN1 and TP53). To further illustrate the potential pleiotropy and drug side effects of significant druggable genes, we conducted a phenome-wide research of two SNPs associated with two druggable genes of interest (HMGCR and TGFB3). Additionally, we performed enrichment analysis and constructed PPI network for these 21 significant genes to understand the biological significance and interaction mechanisms of these drug targets. Finally, drug prediction and molecular docking were conducted to further validate the pharmaceutical value of these significant druggable genes.

The association between HMGCR and migraine has been supported by multiple prior studies. One study indicated that migraine has significant shared signals with certain lipoprotein subgroups at the HMGCR locus [29]. Hong et al. found that HMGCR genotypes associated with higher LDL cholesterol levels are linked to an increased risk of migraine [30]. Statins inhibit the activity of HMG-CoA reductase, which is encoded by the HMGCR gene, to exert their lipid-lowering effects and have been widely used in the prevention and treatment of coronary heart disease and ischemic stroke. Previous clinical research has shown that simvastatin combined with vitamin D can effectively prevent episodic migraines in adults [31]. Additionally, HMGCR may also be involved in immune modulation, with studies suggesting that migraine patients experience neuroinflammation due to activation of the trigeminal-vascular system, leading to peripheral and central sensitization of pain and triggering migraine attacks [32, 33]. HMGCR inhibitors can suppress the production of inflammatory mediators and cytokines, thus reducing inflammatory responses [34]. We speculate that the role of HMGCR in regulating inflammation and immunity may have influenced the drug prediction results generated by DSigDB, which based on Gene Set Enrichment Analysis (GSEA) [24, 35, 36], diluting the role of HMGCR in regulating lipid metabolism. Therefore, statins did not appear in the predicted list of candidate drugs.

TGFB1 and TGFB3 encodes different secreted ligands of the transforming growth factor-beta (TGF-β) superfamily of proteins, namely TGF-β1 and TGF-β3. TGF-β is a pleiotropic cytokine closely associated with immunity and inflammation [37]. Research indicated that TGF-β3 can inhibit B cell proliferation and antibody production by suppressing the phosphorylation of NF-κB, thus exerting its anti-inflammatory effects [38]. The activation of the classical NF-κB pathway is a key mechanism that upregulates pro-inflammatory cytokines, promoting central sensitization and leading to the onset of chronic migraine [39]. A previous clinical study indicated that the serum levels of TGF-β1 are significantly elevated in migraine patients [40]. Ishizaki et al. found that TGF-β1 levels in the platelet poor plasma of migraine patients are significantly increased during headache-free intervals [41]. Bø et al. discovered that during acute migraine attacks, the levels of TGF-β1 in cerebrospinal fluid are significantly higher compared to the control group [42]. Although some studies consider TGF-β1 to be an anti-inflammatory cytokine [43], based on previous research and the results of this study, we believe that TGFB1 and its encoded protein, TGF-β1, are associated with an increased risk of migraine. The pleiotropic effects of TGF-β1 on inflammation may depend on concentration and environment [44]. In addition, we found an association between TGFB3 and IGF1 in our phenome-wide research. A previous MR study showed that increased levels of IGF1 are causally associated with decreased migraine risk [45]. Recent experimental results suggest that the miR-653-3p/IGF1 axis regulating the AKT/TRPV1 signaling pathway may be a potential pathogenic mechanism for migraine [46]. The beneficial effects of TGF-β3 and IGF1 on migraine may be associated with the regulation of gene expression in different microenvironments to promote the transition of microglial cells from M1 (pathogenic) to M2 (protective) phenotypes [47].

Among the 13 genes targeted by some commonly used migraine treatment drugs, the MR results for 3 genes were significant in blood or brain eQTL. Although only one gene remained significant after FDR correction, this still demonstrates that the significant genes newly identified in this study are reliable and have potential as drug targets to some extent. The lack of significance in certain drug target genes may be related to the insufficient sample size of the migraine GWAS data included in our study. It would be meaningful to validate the results of this study with more large-sample GWAS data available in the future.

In this study, DSigDB predicted 10 potential drugs for migraine, but current clinical research is mainly focused on melatonin and dexamethasone. ClinicalTrials (https://clinicaltrials.gov/) has registered multiple studies on the efficacy of melatonin and dexamethasone for migraine. Many research findings differ differently and controversially. A published clinical study on acute treatment of pediatric migraine showed that both low and high doses of melatonin contributed to pain relief [48]. The consensus published by the Brazilian Headache Society in 2022 lists melatonin as a recommended medication for preventing episodic migraine (Class II; Level C) [49]. However, study indicated that bedtime administration of sustained-release melatonin did not lead to a reduction in migraine attack frequency compared to placebo [50]. Dexamethasone has shown good efficacy for severe acute migraine attacks [51]. The 2016 guidelines for the emergency treatment of acute migraines in adults, issued by the American Headache Society, mention that dexamethasone should be administered to prevent the recurrence of migraine (Should offer—Level B) [52]. But study suggested that dexamethasone does not reduce migraine recurrence [53].

An animal study has shown that clofibrate can improve oxidative stress and neuroinflammation caused by the exaggerated production of lipid peroxidation products [54]. Clofibrate can activate peroxisome-proliferator-activated receptors (PPAR) α, inhibit the activation of the NF-κB signaling pathway and the production of interleukin (IL)-6, exerting an anti-inflammatory effect [55, 56]. Additionally, a recent animal study indicated the upregulation of astrocytic activation and glial fibrillary acidic protein (GFAP) expression in the trigeminal nucleus caudalis (TNC) in migraine mice model induced by recurrent dural infusion of inflammatory soup (IS). This was accompanied by the release of various cytokines, increased neuronal excitability, and promotion of central sensitization processes [57]. Clofibrate can reduce the activation of astrocytes and the expression of GFAP, thereby inhibiting neuroinflammation [54]. Andrographolide is a major bioactive constituent of Andrographis paniculata, has broad effects on various inflammatory and neurological disorders [58–60]. Although we did not find any migraine clinical trials related to clofibrate and andrographolide on PubMed and ClinicalTrials, we believe that the prospects for using clofibrate and andrographolide in the treatment of migraine are quite promising. We hope to see more research on the association of clofibrate and andrographolide with migraine in the future.

Our study has several advantages. First, we provided compelling genetic evidence about migraine drug targets using MR, utilizing the largest publicly available GWAS data to date. Additionally, colocalization analysis helps reduce false negatives and false positives to ensure the robustness of the results. Enrichment analysis and PPI illustrate the functional characteristics and regulatory relationships of these targets genes, providing potential avenues for migraine drug development. The drug predictions demonstrate the medicinal potential of these genes, and high binding activity from molecular docking indicates the strong potential of these genes as drug targets. Our research conducts a comprehensive evaluation from identifying migraine-related druggable genes to drug binding properties, proposing migraine drug targets with compelling evidence.

This study also includes several notable limitations. Firstly, the number of eQTL IVs in MR is limited, with most not exceeding three SNPs, which restricts the credibility of the MR results. Additionally, while MR offers valuable insights into causality, it assumes a linear connection between low-dose drug exposure and the exposure-outcome relationship, which may not fully replicate real-world clinical trials that typically assess high doses of drugs in a short timeframe. Therefore, MR results may not accurately reflect the effect sizes observed in actual clinical settings, nor fully predict the impacts of drugs. Secondly, the generalizability of this study is limited by its primary inclusion of individuals of European descent. Extrapolating the findings to individuals of other genetic ancestry populations requires further research and validation to ensure broader applicability. Thirdly, the study focuses mainly on cis-eQTLs and their relationship with migraine, potentially overlooking other regulatory and environmental factors that contribute to the complexity of the disease. Fourthly, while enrichment analysis is valuable, it has inherent limitations as it relies on predefined gene sets or pathways, which may not encompass all possible biological mechanisms or interactions. A lack of significant enrichment does not necessarily mean there is no biological relevance, and researchers should interpret results cautiously. Fifth, the accuracy of molecular docking analysis largely depends on the quality of the protein structures and ligands. While this method identified potential drug targets, it does not guarantee their efficacy in clinical settings. Subsequent experimental validation and clinical trials are necessary to confirm the therapeutic potential of the identified targets. Moreover, we only investigated the side effects of 2 significant druggable genes. The effects of drugs on targets are very broad, and many off-target effects cannot be explored through MR, requiring further basic and clinical trials to gain a more comprehensive understanding. Finally, the clinical relevance of our study results needs further validation; the lack of clinical data related to our study is a significant limitation.

## Conclusions

This study utilized MR and colocalization analysis to identify 21 potential drug targets for migraine, two of which were significant in both blood and brain. These findings provide promising leads for more effective migraine treatments, potentially reducing drug development costs. The study contributes valuably to the field, highlighting the importance of these druggable genes significantly associated with migraine. Further clinical trials on drugs targeting these genes are necessary in the future.

### Supplementary Information

Supplementary Material 1.

## Data Availability

The Migraine GWAS dataset provided by Hautakangas et al. can be obtained by contacting International Headache Genetics Consortium [5]. Other data can be obtained from the original literature and websites.

## Abbreviations

MR: Mendelian randomization
eQTL: Expression quantitative trait loci
GWAS: Genome-wide association studies
CGRP: Calcitonin gene-related peptide
SNPs: Single nucleotide polymorphisms
IVs: Instrumental variables
DGIdb: Drug-Gene Interaction Database
FDR: False discovery rate
IHGC: International Headache Genetics Consortium
TSS: Transcriptional start site
IVW: Inverse-variance weighted
5-HT: 5-Hydroxytryptamine
GABA: γ-Aminobutyric acid
PP: Posterior probabilities
PheWAS: Phenome-wide association study
GO: Gene Ontology
KEGG: Kyoto Encyclopedia of Genes and Genomes
BP: Biological process
MF: Molecular function
CC: Cellular component
PPI: Protein–protein interaction
DSigDB: Drug Signatures Database
PDB: Protein Data Bank
LDL: Low-density lipoprotein
GSEA: Gene Set Enrichment Analysis
IGF1: Insulin-like growth factor 1
TGB-β: Transforming growth factor-beta
PPAR: Peroxisome-proliferator-activated receptors
IL: Interleukin
GFAP: Glial fibrillary acidic protein
TNC: Trigeminal nucleus caudalis
IS: Inflammatory soup
